# Electricity in Diseases of the Nose, Throat and Ears, and in Some Diseases of the Thoracic and Abdominal Viscera Which Result from Chronic Nasal and Pharyngo-nasal Catarrh*Read before the American Rhinological Association at St. Louis, October, 1884.

**Published:** 1885-03-20

**Authors:** J. R. Van Allen

**Affiliations:** Kansas City, Mo.


### ELECTRICITY IN DISEASES OF THE NOSE, THROAT, AND EARS, AND IN SOME DISEASES OF THE THORACIC AND ABDOMINAL VISCERA WHICH RESULT FROM CHRONIC NASAL AND PHARYNGO-NASAL CATARRH.*

By J. R. Van Allen, M.D., Kansas City, Mo.

For several years I have been testing the efficacy of electricity as a remedial agent, and though at times disappointed in my expectations, at others I have been gratified by its evident power in alleviating diseased conditions promptly and efficaciously. I am convinced that several causes operate to discourage and disappoint many who have made tests of this potent remedy, among which are the exalted expectations of the operator, its untimely application, the undue strength of the currents and the too frequent and too prolonged subjection of the patient to its influence.

In active congestions it should never be employed till turgesence has been relieved, just as we reduce the acute inflammation before excising a tonsil. When we consider that the primary effect of electricity is to exalt temperature and increase circulatory congestion, it is obvious that the above rule should be strictly observed.

It is erroneous to suppose that no benefit is secured unless the patient very sensibly feels the power of the current. Experience proves that the milder the influence, the result being attained, the better it is for the disease and the patient. As there is no mathematical rule regulating the degree nor the term of electrical application, it is necessary to be governed in these particulars, to great extent, by the sensation of the subject, who will indicate the propriety of lessening the force or limiting its use if pain is produced or existing pain increased, with agitation and excitement accompanying rigors and perspiration. Such symptoms indicate that, at

* Read before the American Rhinological Association at St. Louis, October, 1884.

least, the remedy is not beneficial. If, per contra, there is induced a bouyant feeling, followed by soothed sensations, improved appetite and refreshing sleep, confidence in the ultimate curative influence of the measure may be indulged.

Electricity, while certainly a valuable adjuvant, can not be regarded as the only requisite in the treatment of catarrhal troubles, but as it serves to allay pain, arouses the digestive and assimilative processes, improves the appetite and induces sleep, it is evident that it paves the way for local treatment which, from experience, we know to be beneficial. Electricity confers tone upon the system, enabling it to throw off disease, while, by preventing the disposition to readily "take cold," it controls an otherwise constant aggravation of the original malady. With such treatment it is possible to subdue chronic catarrhal inflammation and, at the same time, remedy hypertrophied and ulcerated mucous surfaces. Aggravated cases of chronic nasal and pharyngo-nasal catarrh are characterized by general debility with great dyspnœa, night sweats, cardiac palpitation, cough with free expectoration, headache, cold extremities, variable appetite, and present the appearances of anemia. The vessels of the pharyngeal and nasal cavities are enlarged, the lining membrane thickened, anæsthesia of the entire mucous surface, paresis of the soft palate, aphonia of some degree, impaired sense of taste and smell, difficult deglutition, insomnia, partial deafness, tinnitus aurium, constipation, deranged kidneys and sometimes irritability of the urinary bladder. In females, to this train of symptoms is superadded, leucorrhœa, amenorrhœa or dysmenorrhœa. Without minutely tracing each individual symptom and condition thus indicated to its ultimate cause, or considering it in its anatomical and physiological relations, it remains that all and singly they are based on a general atonic condition which it is necessary to overcome in order to relieve, and this can be more readily accomplished through the stimulant, sedative and tonic properties of electricity scientifically applied than through the slower, more disagreeable and less reliable action of drugs.

This agent may be applied in three different ways, viz.: By central galvanization, which influences the central nervous system, including the pneumogastric; general faradization, which affects all parts of the body; localized electrization, which limits the action to a selected part.

The first two methods are generally adopted, and the application by central galvanization is made by placing the anode to the epigastric region, the cathode first to the frontis and vertex for about five minutes, and afterward to either side of the neck to influence the pneumogastric and cervical sympathetic; continuing this for the same space of time it is then applied to the nuchæ and over the entire vertebral column for ten minutes.

In general faradization the feet rest on a copper plate, the anode immersed in a bowl of warm water, while the cathode is passed along the spine and over any part to be especially treated, or perhaps a better plan is to make use of both currents, applying each for ten or fifteen minutes. General faradization acts similarly to *massage*, and is, like it, at times of signal servive.

Electricity has been found beneficial in the treatment of dyspepsia, a condition frequently complicating our cases. It is best treated by central galvanization, but in connection with general faradization, a more marked curative effect is obtained. And, not only can dyspepsia be in this manner relieved, but its congener, constipation, can be overcome by extending the application over the abdominal surface.

Aphonia can generally be benefitted by placing the anode over the sternum or at the nuchæ, the cathode being passed over the larynx or directly in contact with it.

Tinnitus aurium has been signally relieved by galvanization. The patient holds the anode in the hand opposite to the ear under treatment, the cathode is applied to the tragus or in the auditory canal, first filling it with warm salt water. The application should not be continued for more than fifteen seconds, with a mild current which should be very gradually broken by drawing the anode from the tips of the fingers. If a sound of lower tone be produced, arrest the treatment till the next sitting. The electrodes should invariably be placed in position before starting the battery.

For loss of taste, use central galvanization by the direct application of the cathode to the tongue and the anode to the ensiform cartilage; for loss of smell, use central galvanization and localized electrization; for asthma, due to paresis of nerve tissue surrounding the air cells, use central galvanization; for menstrual troubles or prolapsus uteri, place the anode to the soles and, dividing the cathode, pass one branch over the region of the uterus, the other over the lumbar region, or a positive electrode may be applied to the os uteri. Benefit may be obtained in nasal catarrh by local electrization. Place the anode at the nuchæ and pass the cathode over the nose. In atrophic or dry catarrh apply the cathode to the nasal mucous membrane as a stimulant, exciting secretion. Insomnia can be overcome by central galvanization and general faradization. Paresis of the soft palate is best treated by central galvanization and general faradization. Place the anode over the ensiform cartilage, the cathode to the palate.

In conclusion, I ask for this powerful agent a fair trial, believing from the many excellent results already recorded that it is destined to play an important part in the future treatment of disease.—*St. Louis Med. College.*

---

**The Treatment of Sick Headache.**—Dr. W. Gill Wylie, of New York, has produced excellent results with the following method of treatment: So soon as the first pain is felt, the patient is to take a pill, or capsule, containing one grain of inspissated ox-gall and one drop of oil of gaultheria, every hour until relief is felt, or until six have been taken. Dr. Wylie states that sick headache, as such, is almost invariably cut short by this plan, although some pain of a neuralgic character remains in a few cases.—*New York Medical Journal.*

THE COMMA BACILLUS—A Koch sure thing.—*Med. Rec.*

